# Perfect Milk

**Published:** 1877-12

**Authors:** 


### *PERFECT MILK.*

Our friends of the Health Food Company announce that they have perfected arrangements for the daily delivery of pure Alderney milk to consumers in all parts of the city. Physicians, and all who estimate pure and perfect milk at its true value, will be rejoiced to be made acquainted with this fact. The milk is produced in Litchfield County, Connecticut, a locality which is celebrated for its fine Alderney stock, and is sent to the consumers in sealed vessels, which cannot be tampered with or violated without detection. The advertisement of the company on another page fully explains the matter, and we only allude to it for the purpose of bearing cordial testimony to the honesty and faithfulness, and singleness of purpose of the gentlemen who have inaugurated this difficult enterprise. Pure and perfect milk has never before been offered for sale in New York. Alderney milk is sent to a few wealthy families from friends in the country, but other city people have never seen it here until now. The wretched poisonous fluid sold for milk among us, kills more babies and invalids than all other causes combined. The trouble is not merely that milk is watered ; that is a wicked cheat, but it does not effect the destruction of many lives. The great injury comes from the terrible stuff which the milk-producing cows are fed upon—the putrefactive refuse of distilleries and breweries. That this is the chief source of sickness and death among delicate infants in New York, we unhesitatingly assert, and we make the statement the more freely, because a means of escape is now provided by thoroughly trustworthy parties.

